# A rapid and cost-effective method for genotyping apolipoprotein E gene polymorphism

**DOI:** 10.1186/s13024-016-0069-4

**Published:** 2016-01-12

**Authors:** Li Zhong, Yong-Zhuang Xie, Tian-Tian Cao, Zongqi Wang, Tingting Wang, Xinxiu Li, Rui-Chi Shen, Huaxi Xu, Guojun Bu, Xiao-Fen Chen

**Affiliations:** Fujian Provincial Key Laboratory of Neurodegenerative Disease and Aging Research, Institute of Neuroscience, Medical College, Xiamen University, Xiamen, 361102 China; Fujian Institute of Subtropical Botany, Xiamen, 361006 China; Department of Neuroscience, Mayo Clinic, Jacksonville, FL 32224 USA

**Keywords:** Apolipoprotein E, *APOE* genotype, Real Time PCR, Sequencing, TaqMan, Polymorphism

## Abstract

**Background:**

Apolipoprotein E (ApoE) is a major cholesterol carrier and plays an important role in maintaining lipid homeostasis both in the periphery and brain. Human *APOE* gene is polymorphic at two single nucleotides (rs429358 and rs7412) resulting in three different alleles (ε2, ε3 and ε4). ApoE isoforms modulate the risk for a variety of vascular and neurodegenerative diseases; thus, *APOE* genotyping is crucial for predicting disease risk and designing individualized therapy based on *APOE* genotype.

**Results:**

We have developed an *APOE* genotyping method that is based on allele-specific PCR methodology adapted to Real Time PCR monitored by TaqMan probe. Rather than using TaqMan probes specific for the two polymorphic sites, only one TaqMan probe is used as the polymorphic alleles are recognized by site-specific PCR primers. Each genotyping assay can be completed within 90 minutes and is applicable to high-throughput analysis. Using this protocol, we genotyped a total of 1158 human DNA samples and obtained a 100 % concordance with the *APOE* genotype determined by sequencing analysis.

**Conclusion:**

The *APOE* genotyping assay we have developed is accurate and cost-effective. In addition, our assay can readily be applied to genotyping large sample numbers. Therefore, our *APOE* genotyping method can be used for assessing the risk for a variety of vascular and neurodegenerative diseases that have been reported to be associated with *APOE* polymorphism.

## Background

The human apolipoprotein E (apoE) gene is mapped to chromosome 19q13.2, which consists of four exons and three introns [1]. *APOE* gene is polymorphic at two single nucleotides (rs429358 and rs7412), resulting in three different alleles (ε2, ε3 and ε4) and six *APOE* genotypes (ε2/ε2, ε2/ε3, ε2/ε4, ε3/ε3, ε3/ε4 and ε4/ε4) [2]. The worldwide frequency of ε2, ε3 and ε4 allele is 8.4 %, 77.9 % and 13.7 %, respectively [3, 4]. However, the *APOE* allele frequencies vary widely among different ethnic populations [5]. Differences among the three apoE isoforms reside in the amino acid residues 112 and 158, where either cysteine or arginine is present: E2 (Cys 112, Cys 158), E3 (Cys 112, Arg 158), and E4 (Arg 112, Arg 158) [6]. Despite differences by only one or two amino acids, the structural and functional differences among the three apoE isoforms can be profound to affect disease risk [7, 8].

ApoE is a major cholesterol carrier and plays an important role in maintaining lipid homeostasis both in the periphery and brain [3, 6]. In the periphery, apoE is synthesized predominantly by liver and macrophages. In the brain, apoE is produced primarily by astrocytes and delivers cholesterol and other essential lipids to neurons through members of the low-density lipoprotein receptor (LDLR) family [9–12]. The single amino acid differences among the three apoE isoforms alter the protein’s structure and influence its lipid association and receptor binding; therefore apoE modulates cholesterol homeostasis in an isoform-dependent manner [13]. Notably, apoE2 binds to LDLR with ~50-fold weaker affinity than apoE3 and apoE4. As a result, apoE2 transports lipids less efficiently, and ε2 homozygosity is associated with an increased risk for type III hyperlipoproteinemia [14–16]. ApoE4 preferentially binds to large lipoprotein particles and is associated with increased risk for hypercholesterolemia and atherosclerosis, faster HIV disease progression, and accelerated telomere shortening [3, 17, 18].

Most importantly, genome-wide association studies have confirmed that the ε4 allele of *APOE* is the strongest genetic risk factor for late-onset Alzheimer’s disease (LOAD) [9, 19–21]. Compared with those with an ε3/ε3 genotype, the risk of AD was increased in individuals with one copy (ε2/ε4, OR 2.6; ε3/ε4, OR 3.2) or two copies (ε4/ε4, OR 14.9) of the ε4 allele [4, 22, 23]. Conversely, the ε2 allele of *APOE* has a protective effect against AD [24]. The risk of AD in individuals carrying an ε2/ε2 (OR 0.6) or ε2/ε3 (OR 0.6) genotype is lower than those carrying ε3/ε3 [4]. Additionally, the ε4 allele of *APOE* was found to be a risk factor for other neurodegenerative diseases including cerebral amyloid angiopathy (CAA) [25], dementia with Lewy bodies [26, 27] and multiple sclerosis [28].

As *APOE* genotype predicts the risk for a variety of vascular and neurodegenerative diseases, it is critical to develop rapid and cost-effective methods to analyze *APOE* gene polymorphism. Several different *APOE* genotyping methods have been developed. Among them, the PCR-Restriction Fragment Length Polymorphism (PCR-RFLP) analysis is a conventional method applied to genotype *APOE* polymorphism [29, 30]. However, this method is complex and labor-intensive as it involves multiple steps including restriction enzyme digestion. PCR plus sequencing or mass spectrometry is an effective method, but requires expensive detection equipment and is also labor-intensive [31]. Several Real Time PCR-based techniques have been developed to genotype *APOE* gene, including HRM (high resolution melt) [32, 33], TaqMan probe [34] and FRET (Fluorescent Resonance Energy Transfer) [35]. However, the formation of primer-dimers may complicate the melting curves interpretation, and the use of FRET and multiple TaqMan probes is in general costly.

In view of the importance of *APOE* genotyping in predicting individual risk for a variety of vascular and neurodegenerative diseases, we have developed a rapid and cost-effective method for analyzing *APOE* polymorphism. Using this protocol, we genotyped a total of 1158 human DNA samples and obtained a 100 % concordance with the *APOE* genotype determined by sequencing analysis. Therefore, the method we have developed for *APOE* genotyping is precise and suitable for genotyping large sample cohorts.

## Results

### *APOE* genotyping assay development and validation

**Fig. 1:**
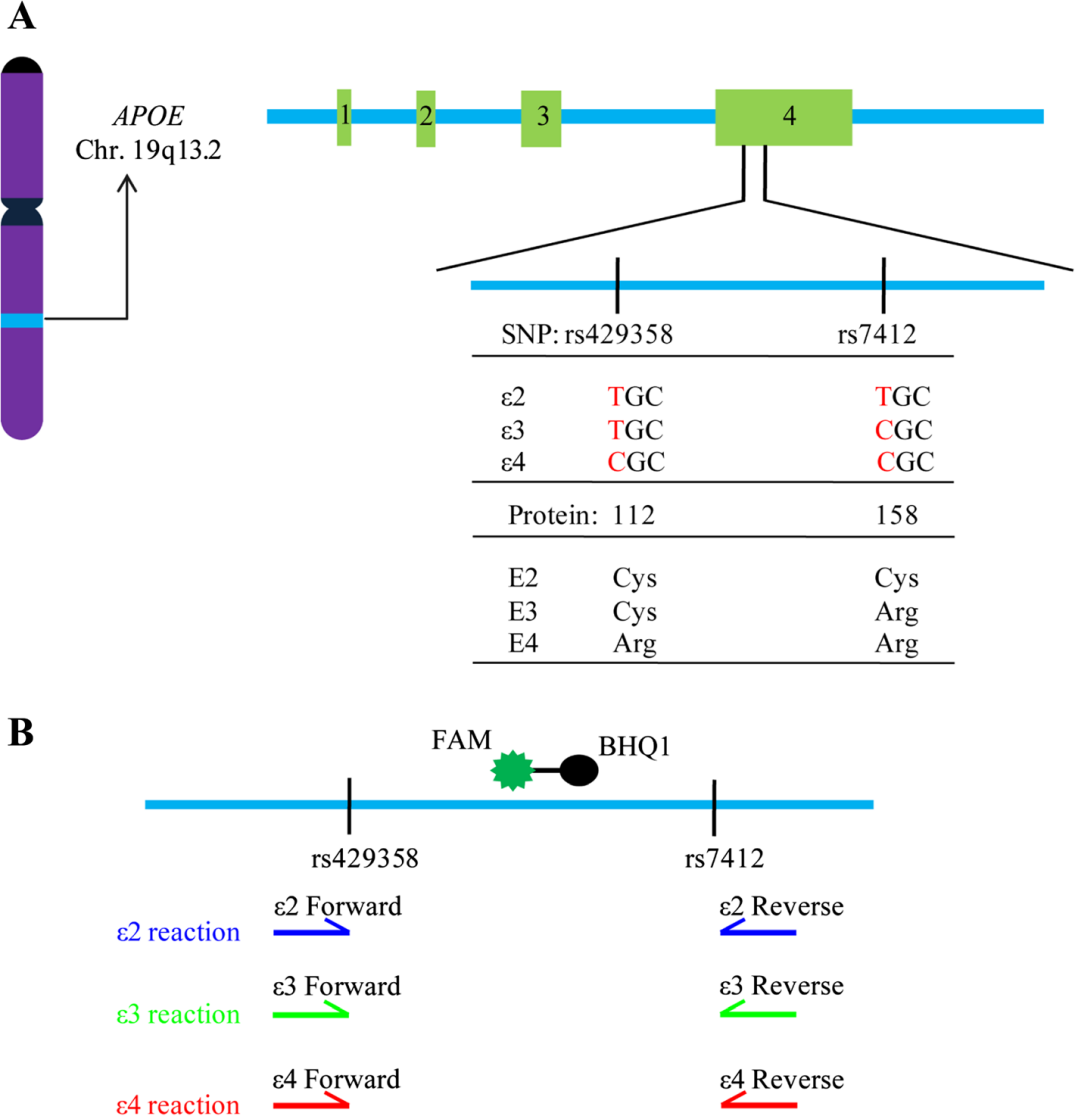
Schematic diagram of the human *APOE* gene and *APOE* genotyping method. **a** The *APOE* gene is located on chromosome 19, and is polymorphic at two single nucleotides (rs429358 and rs7412) resulting in three different alleles (ε2, ε3 and ε4). **b** Amplifications of the *APOE* ε2, ε3 and ε4 alleles are initiated by allele-specific PCR primers. One double-dye oligonucleotide TaqMan probe was included in all reactions to monitor real-time DNA amplification products

Our assay was based on allele-specific PCR methodology adapted to Real Time PCR monitored by TaqMan probe. Initial PCR primers were designed according to the nucleotide differences located at the two SNPs within exon 4 of the *APOE* gene, rs429358 and rs7412 (Fig. 1a). We screened a group of oligonucleotide primers and obtained three pairs that gave specific amplifications of ε2, ε3 and ε4 allele, respectively (Table 1). In order to monitor real-time DNA amplification products, one double-dye oligonucleotide TaqMan probe was included in all reactions. The probe has a FAM fluorophore attached to its 5’ end and a BHQ quencher molecule attached to its 3’ end (Fig. 1b). The probe initially hybridizes to the template strand via its complementary sequences. Upon PCR amplification, Taq polymerase will degrade the probe during strand extension, resulting in the separation of fluorophore and the quencher which allows the excitation of the fluorophore by laser [36].

**Table 1:** Sequences of primers and probes for the *APOE* genotyping assay

Name	Sequence (5’-3’)
ε2-Forward	GCGGACATGGAGGACGTGT
ε2-Reverse	CCTGGTACACTGCCAGGCA
ε3-Forward	CGGACATGGAGGACGTGT
ε3-Reverse	CTGGTACACTGCCAGGCG
ε4-Forward	CGGACATGGAGGACGTGC
ε4-Reverse	CTGGTACACTGCCAGGCG
*APOE* probe	FAM-CAGCTCCTCGGTGCTCTGGC-BHQ1
*ACTB*-Forward	GACGTGGACATCCGCAAAGAC
*ACTB*-Reverse	CAGGTCAGCTCAGGCAGGAA
*ACTB* probe	HEX-TGCTGTCTGGCGGCACCACCATGTACC-BHQ1

**Fig. 2:**
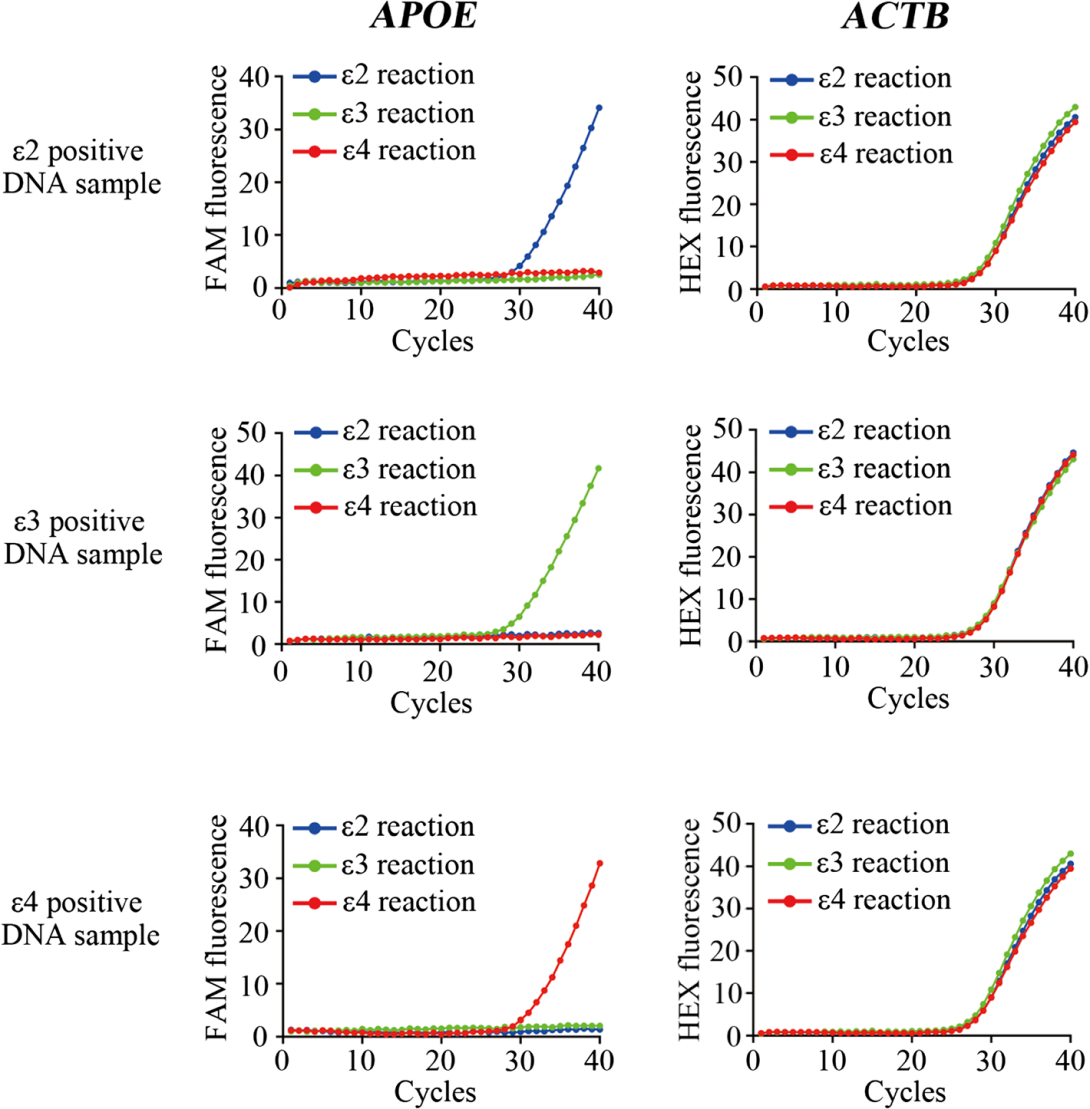
*APOE* genotyping on ε2-, ε3- or ε4-positive plasmid DNA by Real Time PCR. Representative amplification curves for *APOE* and *ACTB* are shown. 12000 copies of each plasmid DNA are used as templates. Blue: ε2 reaction; Green: ε3 reaction; Red: ε4 reaction. FAM fluorescence: *APOE* gene; HEX fluorescence: *ACTB* gene

To validate the efficiency and specificity of our assay, we synthesized three 197-bp DNA sequences covering the two SNPs for ε2, ε3 or ε4 allele and a 179-bp DNA sequence of beta-actin gene (*ACTB*) which serve as positive controls for *APOE* genotyping. The TaqMan probe for *ACTB* was designed similar to *APOE* but the former has a HEX fluorophore attached to its 5’ end. Genotyping of each sample was carried out in parallel reactions, namely ε2, ε3 or ε4 reaction. Each reaction contains allele-specific primers for *APOE* in combination with the primers for *ACTB*. Specific amplification curve appeared only in ε2 reaction when ε2-positive DNA was used (Fig. 2). Similar results were observed in ε3 or ε4 reaction, demonstrating the specificity of our detecting system.

### *APOE* genotyping of clinical DNA samples

**Fig. 3:**
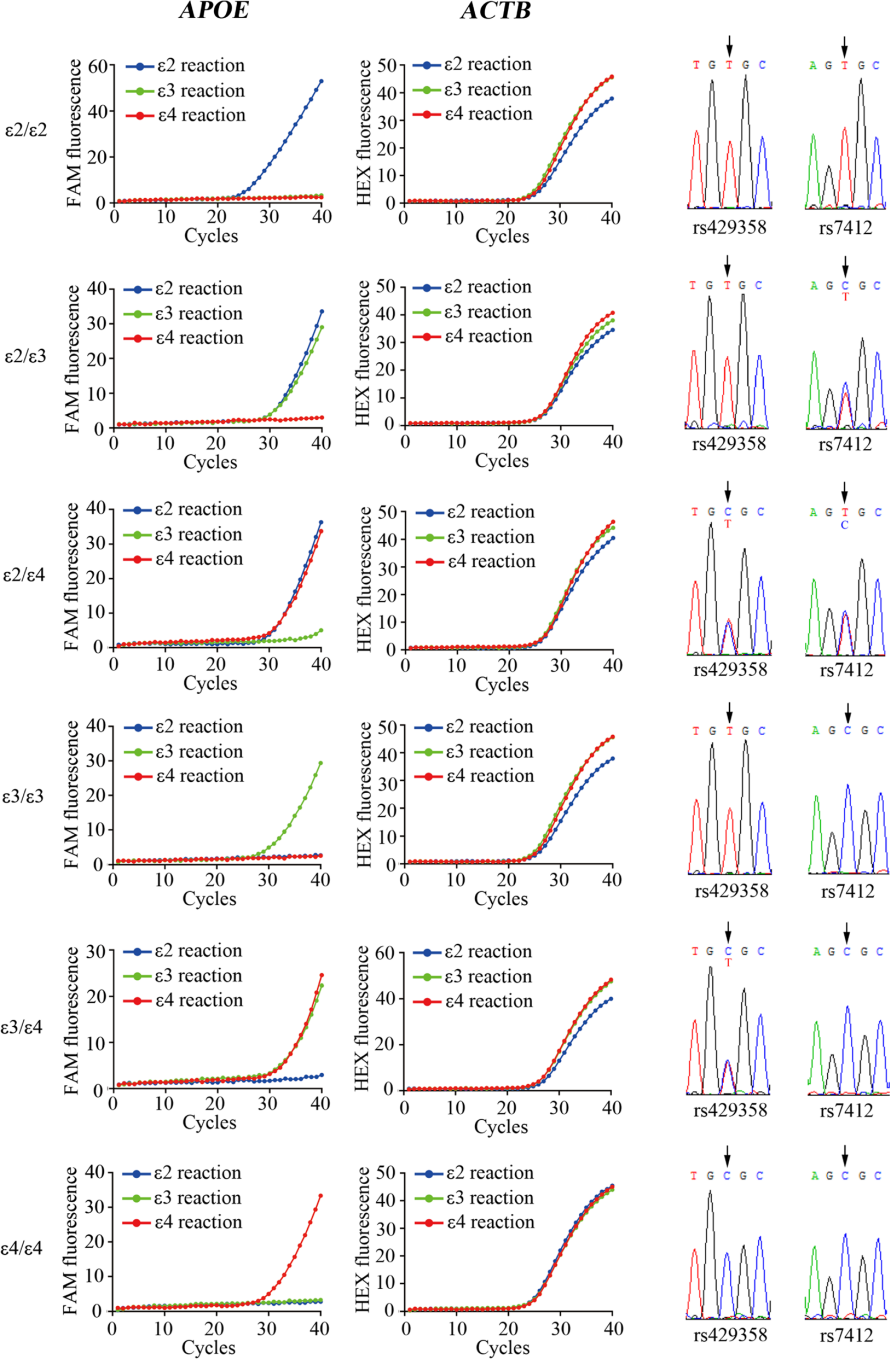
*APOE* genotyping on clinical DNA samples by Real Time PCR and DNA sequencing. Representative amplification curves for *APOE* and *ACTB*, and representative sequencing results for the two SNPs (rs429358 and rs7412) are shown. Blue: ε2 reaction; Green: ε3 reaction; Red: ε4 reaction. FAM fluorescence: *APOE* gene; HEX fluorescence: *ACTB* gene

We further validated our *APOE* genotyping assay using human DNA samples. Genomic DNA was extracted from 1158 clinical blood samples and undergone subsequent *APOE* genotyping analysis. The presence/absence of haplotypes was either determined by differential amplification with the three specific amplification setups for ε2, ε3 or ε4 allele, or by DNA sequencing performed by Sangon Biotech using the ABI 3730XL DNA Sequencer. In the clinical sample testing, our *APOE* genotyping assay has been efficient such that a single Real Time PCR reaction took approximately 90 minutes, and the Roche LightCycler 480 II system has the potential to run 384 reactions at one time. Typical results are presented in Fig. 3, with genotyping results and allele frequencies summarized in Table 2. *APOE* genotyping using our assay showed 100 % concordance with DNA sequencing results, demonstrating the accuracy and reliability of our protocol. The *APOE* allele frequency for ε2, ε3 and ε4 in our Chinese Han population was 7.90 %, 83.94 %, 8.16 %, respectively. Thus, the frequency of the ε4 allele was lower than that for the world-wide population (8.4 %, 77.9 % and 13.7 % for ε2, ε3 and ε4, respectively), but was similar to previous studies in Chinese population [4, 5, 37]. To provide guidance on evaluating the accuracy of our *APOE* genotyping method, receiver operating characteristic (ROC) curve analysis was performed to assess the cut-off ΔCt values for each Real Time PCR reaction (Table 3). A sample is considered to be negative for the corresponding genotype analysis if the ΔCt value is higher than the cut-off value.

**Table 2:** Analysis of *APOE* genotypes and allele frequency in Chinese population

Cohort	No.	*APOE* genotypes (No.)						Accuracy (%)	Alleles (%)		
		ε2/ε2	ε2/ε3	ε2/ε4	ε3/ε3	ε3/ε4	ε4/ε4		ε2	ε3	ε4
Zhongshan	399	2	60	6	278	51	2	100	8.77	83.58	7.65
Fujian	390	2	50	5	271	60	2	100	7.56	83.59	8.85
Huadong	369	3	43	5	265	52	1	100	7.32	84.69	7.99
Total	1158	7	153	16	814	163	5	100	7.90	83.94	8.16

Genomic DNA was extracted from peripheral blood samples obtained from three hospitals (399 samples from Zhongshan Hospital Affiliated to Xiamen University, 390 samples from Fujian Medical University Union Hospital, and 369 samples from Huadong Hospital Affiliated to Fudan University). The *APOE* genotypes and allele frequency were analyzed by both Real Time PCR and DNA sequencing. The 100 % accuracy was defined when *APOE* genotyping using the Real Time PCR assay showed 100 % concordance with DNA sequencing results

**Table 3:** Cut-off values for ΔCt calculated by ROC curve analysis

Reactions	Cut-off values
ε2 reaction	9.2
ε3 reaction	10.4
ε4 reaction	11.1

The cut-off ΔCt values for the three reactions were calculated from ROC curve analysis, which represent the threshold cycle above which a sample is considered to be negative for the corresponding genotype analysis

## Discussion

We have developed an *APOE* genotyping assay that is based on allele-specific PCR methodology adapted to Real Time PCR monitored by a common TaqMan probe. Each genotyping analysis can be accomplished within 90 minutes and is applicable to high-throughput analysis. We validated the specificity and robustness of our assay in 1158 clinical DNA samples by comparing the results with those from DNA sequencing. All samples genotyped using our assay showed perfect concordance with the *APOE* genotypes determined by sequencing analysis. Therefore, our method for *APOE* genotyping is rapid, precise and cost-effective, with the potential for high-throughput application.

*APOE* gene polymorphism modulates the risk for a variety of vascular and neurodegenerative diseases; thus, *APOE* genotyping is crucial for predicting disease risk and designing individualized therapy based on *APOE* genotype. Recent studies have suggested that therapeutic interventions applied earlier in the course of AD might be more likely to achieve disease modification. Indeed, there is a growing recognition that the pathophysiological process of AD begins many years prior to the onset of clinical symptoms [38]. *APOE* ε4 allele is the strongest genetic risk factor for AD. The mean age of onset and frequency of AD are 68 years and 91 % in ε4 homozygotes, 76 years and 47 % in ε4 heterozygotes, 84 years and 20 % in ε4 non-carriers [39]. Intriguingly, apoE4 has been associated with greater efficacy in at least two clinical trials on mild cognitive impairment [40, 41]. Taken together, *APOE* genotype status may add predictive value to the clinical diagnosis and evaluation of treatment efficacy [8, 42]. Developing an accurate and reliable method for *APOE* genotyping is therefore crucial for AD diagnosis and therapy.

PCR-RFLP analysis is a conventional method for *APOE* genotyping, but is relatively error-prone and labor-intensive due to a number of reaction steps [29, 30]. Compared with the PCR-RFLP method, the accuracy of our *APOE* genotyping method was improved by reducing the steps to one PCR reaction in closed PCR tubes and with no post-PCR sample handling. DNA sequencing is an accurate method for *APOE* genotyping, but it is labor-intensive and not suitable for high-throughput analysis. Our assay was based on allele-specific PCR methodology and possesses the potential for high-throughput application. Several Real Time PCR-based techniques have been developed to genotype *APOE* alleles, but the formation of primer-dimers makes the interpretation of the melting curves at times difficult [32, 33]. In our protocol, we provide guidance on threshold selection to evaluate the performance of PCR amplification by ROC analysis; therefore, the sensitivity and specificity of our protocol are well defined. TaqMan systems for *APOE* genotyping have been developed for the single nucleotide polymorphisms at rs429358 and rs7412 [34, 43]. However, most protocols require the use of four costly TaqMan probes. Only one TaqMan probe is used in our method as the polymorphic alleles are recognized by site-specific PCR primers. Thus, our method has the potential to design high-throughput application in a way that is cost-effective.

## Conclusions

In this work, we present an *APOE* genotyping method that is accurate and cost-effective. In addition, our assay is based on allele-specific PCR methodology; therefore, can readily be applied to high-throughput *APOE* genotyping. Our *APOE* genotyping protocol can be used in addressing the impact of *APOE* polymorphism on disease risk, and notably in clinical assessments that predict the risk for a variety of vascular and neurodegenerative diseases.

## Methods

### Subjects

A total of 1158 peripheral blood samples were collected from the clinical laboratories in three hospitals (399 samples from Zhongshan Hospital Affiliated to Xiamen University, 390 samples from Fujian Medical University Union Hospital, and 369 samples from Huadong Hospital Affiliated to Fudan University). The study was performed in accordance with the *Declaration of Helsinki* and approved by the Ethics Committees of the three hospitals.

### DNA constructs and reagents

All PCR primers and TaqMan probes were synthesized and purified by Life Technologies (Table 1). Three 197-bp DNA sequences covering the two SNPs for *APOE* ε2, ε3 or ε4 allele and a 179-bp DNA sequence of beta-actin gene (*ACTB*) were synthesized and cloned into pUC57 vector, which served as positive control DNA templates (Sangon Biotech, Shanghai, China). All constructs were verified by DNA sequencing (Sangon Biotech, Shanghai, China). Premix PrimeSTAR HS (R040A) was purchased from TAKARA; TaqMan® Genotyping Master Mix (4371357) was purchased from Applied Biosystems; DMSO (D2650) was purchased from Sigma; and blood DNA extraction kit (DP348-03) was purchased from TIANGEN (TIANGEN, Beijing, China).

### Human genomic DNA isolation

Genomic DNA was extracted from 400 μL peripheral blood by using the blood DNA extraction kit (TIANGEN) according to the manufacturer’s instructions. DNA was diluted with nuclease free water to 8 ng/μL for *APOE* genotyping analysis.

### *APOE* genotyping by Real Time PCR

*APOE* genotyping by Real Time PCR includes three reactions: ε2 reaction (primers ε2-Forward and ε2-Reverse), ε3 reaction (primers ε3-Forward and ε3-Reverse) and ε4 reaction (primers ε4-Forward and ε4-Reverse). Each PCR reaction mixture (15 μL) contained the following reagents: 1 × TaqMan® Genotyping Master Mix, 0.5 μM of each *APOE* primer and *APOE* probe, 0.1 μM of each *ACTB* primer and *ACTB* probe, 40 ng of genomic DNA. Positive control DNA template (ε2, ε3, ε4 plasmid DNA) and negative control (DNA/RNA-free water) were included in each panel of genotyping. The PCR amplification protocol was as follows: Initial activation of AmpliTaq Gold DNA Polymerase at 95 °C for 10 min, followed by 40 cycles with denaturation at 95 °C for 15 sec, and annealing/extension at 64 °C for 1 min. The fluorescence signals were collected during the annealing/extension step. FAM signal indicates *APOE* alleles and HEX signal indicates *ACTB* gene (internal control). The amplification was performed by using the Roche LightCycler 480 II system (Roche).

### *APOE* genotyping by sequencing

For validation purpose, results by the above-mentioned *APOE* genotyping assay were compared with those from DNA sequencing analysis. Briefly, *APOE* gene fragments encompassing the two SNPs were amplified. The amplification reaction was carried out in a volume of 50 μL, which cotains 1 × Premix PrimeSTAR HS, 0.2 μM of each primer (Forward primer: 5’-AGCCCTTCTCCCCGCCTCCCACTGT-3’ and Reverse primer: 5’-CTCCGCCACCTGCTCCTTCACCTCG-3’), 5 % DMSO and 40 ng genomic DNA. The PCR cycling conditions were as follows: Initial denaturation at 98 °C for 4 min followed by 35 cycles with denaturation at 98 °C for 10 sec, annealing at 60 °C for 30 sec, extension at 72 °C for 40 sec; then a final extension at 72 °C for 10 min. All PCR products were purified and sequenced by Sangon Biotech using the ABI 3730XL DNA Sequencer (Applied Biosystems).

### Ct cut-off values calculated by ROC curve analysis

The *APOE* genotypes of 114 human genomic DNA samples were determined either by our *APOE* genotyping method or by DNA sequencing. Receiver operating characteristic (ROC) curve analysis was performed to calculate the cut-off values for ΔCt (calculated by subtracting the Ct value of HEX signal from the Ct value of FAM signal) in our *APOE* genotyping assay. If no amplification curve appeared in the *APOE* allele-specific reaction, the Ct value was considered as 40 for the calculation of ΔCt. The ΔCt values of the ε2/ε3/ε4 reaction were 9.2, 10.4 and 11.1, respectively, as calculated by ROC curve analysis using SPSS software (Table 3).

## Abbreviations

AD: Alzheimer’s disease
ApoE: Apolipoprotein E
CAA: cerebral amyloid angiopathy
Ct: cycle threshold
FAM: carboxyfluorescein
FRET: fluorescent resonance energy transfer
HEX: hexachloro fluorescein
HRM: high resolution melt
LDLR: low-density lipoprotein receptor
LOAD: late-onset Alzheimer’s disease
RFLP: restriction fragment length polymorphism
ROC: receiver operating characteristic
SNP: single nucleotide polymorphism
